# Telemedicine Patient Satisfaction and Cost: A Comparative Study in the COVID-19 Era

**DOI:** 10.7759/cureus.30671

**Published:** 2022-10-25

**Authors:** Abdullah T Alanazi, Bandar Al Hader

**Affiliations:** 1 Health informatics, King Saud Ibn Abdulaziz University for Health Sciences, Riyadh, SAU; 2 Health Sciences, King Abdullah International Medical Research Center, Riyadh, SAU; 3 Health Informatics, King Saud Ibn Abdulaziz University for Health Sciences, Riyadh, SAU; 4 Health Sciences & Research, King Abdullah International Medical Research Center, Riyadh, SAU

**Keywords:** access to health care, cost assessment, efficency, perception towards telemedicine, telemedicine services

## Abstract

Introduction: Telemedicine has the potential to enhance access to healthcare services across distances and this was of great value during the COVID-19 pandemic. However, concerns about user satisfaction in comparison to face-to-face (FTF) interaction remain inadequately addressed.

Objective: This study aimed to assess the impact of telemedicine technology from the patient perspective by comparing patient satisfaction and cost savings of follow-up visits through telemedicine with those of FTF clinical visits. Methods: A cross-sectional study was conducted with two groups i.e., a telemedicine group and a control group that received FTF care at a tertiary hospital. Results: This study demonstrated that the telemedicine group had patient satisfaction levels similar to those of the FTF clinical visit group, having a lower cost of care. Factors associated with patient satisfaction with telemedicine clinical care are identified and discussed. Both groups showed similar results in overall satisfaction with substantial cost savings for telemedicine patients. Conclusion: Positive impacts of telemedicine technology from the patient perspective were seen and decision-makers must expand telemedicine efforts and continuously monitor patient perceptions toward telemedicine.

## Introduction

Access to the healthcare system is a right for every citizen in Saudi Arabia, regardless of whether the citizen lives in a rural or urban area. According to basic Saudi law, article 31, "the State shall protect public health and provide healthcare to every citizen" [1]. However, individuals living in communities in rural areas are underserved, and their general health status is vulnerable. The discrepancies that exist between healthcare services in rural and urban areas regarding the management of chronic diseases, epilepsy, liver transplantation, heart attacks, strokes, cancer, diabetes, and traumatic injuries that exist because they require a comprehensive set of healthcare services are unwarranted and are not inevitable [2]. Furthermore, COVID-19 has impacted people’s mobility, even in normal circumstances; travel to the nearest hospital may impact immediate access to medical interventions, particularly in life-threatening situations. The availability of telemedicine services has brought change and disrupted the delivery of medical services for both rural and urban communities [2]. This study aims to assess the impact of telemedicine technology from a patient perspective by comparing patient satisfaction and cost savings of follow-up visits using telemedicine with those of traditional face-to-face (FTF) clinical visits.

Background and literature review

Telemedicine has particular potential in Saudi Arabia, as the country has experienced rapid population growth, and considering its vast size and the high demand for health services, a discrepancy in accessibility has been observed regarding some healthcare services, particularly those requiring a high level of skill. In addition to the lockdown due to COVID-19, the lack of widespread adoption of health information technology has further burdened healthcare services in Saudi Arabia [3]. Thus, requests for consultation or follow-up appointments at referral hospitals, for example, require patients living in rural areas to travel to major cities [4]. Telemedicine is a technology for providing and sharing health data and clinical expertise over a distance. It has the potential to increase access to high-quality care and decrease costs by eliminating the need for travel to access specific clinical expertise. Furthermore, it may facilitate professional education [5]. However, telemedicine may result in a breakdown in privacy and requires a certain level of information technology (IT) infrastructure and regulations [6 ]. Despite the potential of telemedicine to enhance healthcare outcomes, concerns about its user-friendliness and user satisfaction remain unfathomable and inadequately addressed.

User satisfaction is an indicator of the successful adoption and use of healthcare technologies. A better understanding of user attitudes has a significant role in the perceived usability of services and their anticipated outcomes [3]. A satisfied patient is more willing to follow medical instructions than one who is less satisfied, which results in better clinical outcomes, more rapid recovery, and reduced hospitalization time [7].

Assessing patient satisfaction has become an important indicator of the quality and performance of healthcare services, particularly from the contemporary perspective of healthcare as a patient-centered and consumer-driven system [7]. A study conducted by Abrams et al. found a high level of patient satisfaction with telemedicine visits used for prenatal genetic counseling consultations [8]. Around 80% of the respondents were satisfied as per the study conducted by Lopez et al. [9]. According to a study by Allen et al. [10], the use of telemedicine in oncology provided sufficiently high patient satisfaction “… to warrant further investigation of this modality in the care of rural cancer patients with limited access to cancer specialists." In the current study, patient satisfaction toward telemedicine was assessed in terms of telemedicine quality of care and cost savings. Few studies in Saudi Arabia have been conducted to assess patient satisfaction and how telemedicine might impact patient satisfaction levels. Therefore, this study should add to this body of knowledge and provide insights, particularly regarding recent transformations, as the country strives to develop a more interoperable healthcare system (Vision 2030) [11].

## Materials and methods

This study was a descriptive cross-sectional survey approved by the King Abdullah International Medical Research Center (KAIMRC) (approval no.: SP18R-097). This study aimed to identify the factors associated with patients’ satisfaction and patient cost at a tertiary healthcare facility telemedicine clinic and in remote hospitals connected through telemedicine outreach clinics across Saudi Arabia. Further, physician-patient interaction in FTF and telemedicine sessions was assessed and compared. 

The participants of the study were selected on a convenience basis and were included if they had symptoms or diseases requiring a follow-up visit that could appropriately be conducted using telemedicine. Patients who required a physical examination or who had a residence less than 90 km from the clinic were excluded. Further, patients under the age of 18 were excluded unless they were accompanied by an adult. The study group included patients from rural areas having telemedicine appointments with the tertiary healthcare facility. The control group included patients from rural areas not using telemedicine for their appointments. The control patients were required to travel to the tertiary healthcare facility in Riyadh, Saudi Arabia for an FTF clinic visit. Both groups were given a survey to complete after the purpose of the study was explained by the researcher.

The survey was developed based on an extensive literature review and contains questions on various aspects of patient satisfaction. The survey had three sections. 1) A demographic section with general demographic questions with direct, nominal, and ordinal responses. This section has questions about the following: gender, education, age, region, and distance. 2) The cost of visit section with 12 questions about the following aspects: accommodations (hotels and other types), transportation, distance to the clinic, missed work time, travel costs, and overall cost. This section aimed to assess patient costs for both the telemedicine and FTF groups. 3) Patients’ satisfaction section with questions about their satisfaction level for both sessions i.e., telemedicine and FTF. This third section includes nine items to assess different aspects of patient satisfaction, such as communication abilities of patients and providers, care continuity, waiting time, accessibility, and overall satisfaction using a Likert scale with five response choices (1 = strongly agree; 2 = agree; 3 = neutral, or somewhat satisfied; 4 = disagree; 5 = strongly disagree).

The questions were written in plain language and an attempt was made to avoid ambiguity. To test the facial validity of the tool, three students and a member of the health informatics faculty first examined the tool for facial validity. A pilot study was subsequently conducted to verify the validity and reliability of the tool and its use. The questionnaire was evaluated for its flow and ability to maintain the attention and interest of the respondents. The transition from one question to another was rationally smooth as no comments were made on it and similar sets of questions were used in the literature. The reliability of the survey was tested and the yield acceptable measured (alpha is a = .78)

The responses to the various types of questions used included direct, numeric, and ordinal responses. An open-ended question was provided at the end of the survey to obtain additional feedback from both groups. The data were collected by meeting patients after their visits with their providers for the FTF group and via videoconference technology in the telemedicine group.

## Results

Sixty participants were selected who met the study's inclusion criteria. A consent form was provided; three participants refused to participate without providing reasons, two were not interested, one did not complete the survey and abandoned the interview, three participants missed their scheduled appointments, and two patients had technical problems with the telemedicine clinic. The remaining 49 participants in the study were divided into two groups, with 22 in the telemedicine clinic group and 27 in the FTF control group. The participants were 51% male and 49% female; 46.9% were 18 to 50 years old and 35% were older than 50 years of age. An educational level assessment revealed that 28.6% were illiterate; 33% had completed primary school; 31% had completed high school, and 8% had a bachelor’s degree. In the telemedicine group, 27% of participants were from the Northern region; 55% were from the Southern region, and 14% were from the Central region. Regarding distance from the clinic, 55% of the telemedicine group lived within 100 km; 27% between 100 and 200 km, and 18% lived more than 200 km from the clinic. The average cost of transportation by car to the telemedicine clinic was 88.1 Saudi Arabian Riyal (SAR) per visit. Furthermore, the telemedicine clinic visit required less than a five-hour absence from work and did not require an overnight hotel stay. For the FTF clinic group, 29% of the participants were from the Eastern region, 22% were from the Southern region, 15% were from the Northern region, and 7% were from the Alqassim region. Sixty-three percent of participants lived 600 km or more from the clinic; 26% lived between 400 and 600 km away, and 11% lived less than 400 km from the clinic (Table 1).

**Table 1:** Characteristics of the participants

Measure	Item	Number		Percentage %	
Gender	Male	25		51%	
	Female	24		49%	
Education	Illiterate	14		28.6%	
	Primary school	16		32.7%	
	High school	15		30.6%	
	Bachelor’s degree	4		8.2%	
Age (years)	1-18	9		18.4%	
	19-50	23		46.9%	
	> 50	17		34.7%	
Region		Telemedicine		Face-to-face	
	Central	14%		7%	
	Eastern	4%		29%	
	Western	0%		27%	
	South	55%		22%	
	North	27%		15%	
Distance (km)	0-100	12	54.5%	0	0%
	100 - 200	6	27.3%	0	0%
	200-400	4	18.2%	3	11.1%
	400 -600	0	0%	7	25.9%
	> 600	0	0%	17	63.0%

Accordingly, one-third of the participants used cars to travel to the clinic, while the remaining (67%) used air travel. The average cost of transportation was 1700 SAR, ranging, from 400 to 5000 SAR per visit. For the cost of accommodations, 11% of the participants indicated there was no need for an overnight stay, while 26% estimated a cost of 100 to 499 SAR, 33% estimated a cost of 500 to 999 SAR, and 30% paid 1000 to 2000 SAR to stay in a hotel. Regarding absence from work, 44% were absent for four to seven days. Around 22% were absent for two to three days, 30% missed one day, and approximately 4% required less than a day of absence from work. Thus, the average total cost of an FTF clinic visit was 2203 SAR, while the average total cost of a telemedicine clinic visit average was 172 SAR. Table 2 shows the differences between the two groups.

**Table 2:** Differences between the two groups FTF: Face-to-face, SAR: Saudi Arabian riyal

	Items	FTF Clinic	Telemedicine Clinic
	Did you need assistance getting to the clinic?	Yes, relatives	Yes, relatives
	Did you need accommodations?	3.25-days, average	Not applicable
	Did you know or hear about alternative ways to see the doctor, such as the telemedicine clinic?	No	Yes, during the last session with the doctor
	How much would it normally cost you, in total, to come for a traditional clinic visit (transportation, hotel, lost work time, and other expenses)?	2203 SAR, average	173 SAR, average
	How much is the total transportation cost to get to and from the clinic?	1700 SAR	88.1 SAR
	If you must stay overnight in a hotel for the FTF clinic, how much does it cost?	1055 SAR	Not applicable
	Did you use a car to reach the clinic?	33.30%	100%
	Did you take a flight to reach the clinic?	67.70%	0%

Generally, both groups demonstrated a high level of satisfaction with the clinical session. However, 85% of the FTF participants were extremely satisfied, compared to 68% of the telemedicine group; moreover, 11% of the FTF participants were satisfied, compared to 23% of the telemedicine group.

On whether patients had time to talk to their physicians, both groups reported similar results. When queried about whether their physicians were listening, the FTF participants demonstrated a higher level of agreement (92%) than the telemedicine group (82%). Regarding having sufficient clinic time, physician support for patients, and overall satisfaction, the groups showed similar results (Table 3).

**Table 3:** Comparison of telemedicine and face-to-face (FTF) clinic results

Item	FTF		Telemedicine	
Able to talk	Strongly Agree	93%	Strongly Agree	91%
	Agree	7%	Agree	9%
Physician listened	Strongly Agree	92%	Strongly Agree	82%
	Agree	8%	Agree	18%
Had enough time	Strongly Agree	93%	Strongly Agree	91%
	Agree	7%	Agree	9%
Physician gave support and encouragement	Strongly Agree	90%	Strongly Agree	91%
	Agree	10%	Agree	9%
Able to communicate comfortably	Strongly Agree	90%	Strongly Agree	94%
	Agree	10%	Agree	6%
Overall satisfaction	Strongly Agree	89%	Strongly Agree	90%
	Agree	3%	Agree	5%

An independent sample t-test was used to determine whether a significant difference existed between the two groups regarding patient satisfaction and patient cost. No significant difference was observed between the two clinic types in overall satisfaction; telemedicine: median (M) = 1.11, standard deviation (SD) = 0.320; and FTF: M = 1.27, SD = 0.883 with t(47) = 0.884, and p-value more than 0.05 (p = 0.381). Additionally, no significant difference was observed between the two clinics regarding whether the patient was able to talk with their physician (t(47) = 0.661, p = 0.512). Also, no significant differences were observed between the clinics in clinical support, encouragement, sufficient time, or ease of communication; (t (47) = 0.408, p = 0.685; t (47) = 0.417, p = 0.687; and t (47)=1.25, p = 0.215, respectively). Regarding the total cost to patients using either clinic, a significant difference was observed between the telemedicine and FTF clinics for the total cost (M = 3.63, SD = 0.792, and M = 1.50, SD = 0.913, respectively; t(47)=8.74, p = 0.000). Hence, substantial cost saving is observed with the telemedicine group. Significant differences were also observed between the telemedicine and FTF clinics regarding the need to be absent from work and the distance patients lived from the clinics (t(47) = 17.5, p = 0.001 and t(47)=12.1, p = 0.001, respectively), in which more than two-thirds of the FTF clinic patients required to miss work for at least two days. The results suggested that the telemedicine clinic had an effect on total cost, the need for absence from work, and the patient travel distance required to reach the clinic. The results revealed decreased cost, distance, and length of absence from work for the patients who used the telemedicine clinic compared with the patients using the FTF clinic visit.

## Discussion

The telemedicine clinic was more efficient and had comparable patient satisfaction as clearly demonstrated among patients with chronic diseases who required regular follow-up consultations and clinic visits, notably among patients located in rural areas remote from the tertiary hospital. Therefore, a potential exists for telemedicine to serve rural health needs. The results of this study showed that patients in both groups (telemedicine and FTF) were highly satisfied, or at least satisfied, with the medical services, and no major significant differences were observed between the two groups.

Like other studies, we found that a telemedicine clinic is an acceptable option for providing care to patients in remote areas [12]. In their study, Chae et al. showed that a telemedicine clinic improved patient satisfaction and significantly decreased patient travel distance to the clinic [13]. This result is also in agreement with a study conducted by Boles et al. which showed that 88% of patients were very satisfied with the clinical medical session and the number of visits to the dermatology clinic increased substantially [14]. Patients with chronic conditions such as liver transplantation, epilepsy, and fertility issues require routine and frequent follow-up care. [15] Such patients encounter significant costs despite the short time required for their visit. Further, some patients must be absent from work for days, require accommodations, and sustain transportation and time costs, all to attend a 15-minute routine appointment. Telemedicine clinics may minimize all of these costs, as the results have shown. The cost analysis found that the telemedicine clinic patients had highly significant patient cost savings, time savings, and other benefits compared with the patients who used the FTF clinic.

The average telemedicine clinic patient cost savings was approximately 2000 SAR. Furthermore, patients may need to be accompanied on their visits, particularly the elderly and incompetent patients. These patient escorts encounter hardships similar to the patients regarding travel, accommodations, transportation, absences from work, and other expenses. More than 63% of the FTF patients lived more than 600 km from the tertiary hospital, while 54% of the telemedicine patients lived less than 100 km from the telemedicine clinic. In some regions, the tertiary hospital is approximately 1500 km from some cities, such as Hkagel and Tabuk, and patients require a minimum of 2000 SAR for travel costs and 1500 SAR for accommodations (hotel, transportation, and absence from work). Therefore, patients in these regions often miss appointments. Most patients who had their appointment follow-up through the telemedicine clinic were concerned that this alternative method might affect their eligibility for treatment at the tertiary hospital or that they might be discharged. Regarding the care coordinators at the tertiary hospitals (coordinators between the telemedicine clinics and the tertiary hospitals), we found that most patient complaints, patient preparation to see the physician, and the lack of an appropriate place for the patient during the encounter. Regarding privacy and confidentiality, some privacy issues might be raised because some staff might witness or attend the visit without having a real role in the patient’s care. One of the main challenges for coordinators is a lack of organization of the patient's medical record, laboratory results, the entering of the physician’s orders, and the medications dispensed. All these issues have highly important roles in determining patient satisfaction. Therefore, coordinators should receive training to improve their work associated with tertiary hospitals. As the study indicates, a high percentage of routine patients are not aware of telemedicine clinics or know very little. The patient education departments should educate and promote awareness about the possibility and potential of telemedicine clinics, including complete information on what constitutes a telemedicine clinic, for whose benefit they exist, their advantages and disadvantages, and how they are used.

Study limitations

The results of this study were specific to the context of one site in Saudi Arabia and the costs and patient satisfaction factors may depend on the specific characteristics of Saudi patients. Furthermore, the small number of participants may also limit the generalizability of the findings. Another limitation is that information obtained during interviews may depend on what the patient was willing to share.

## Conclusions

Telemedicine has great potential for patients who live in rural and underserved areas. Telemedicine clinics appear to have significant results in terms of patient cost savings, particularly travel costs and accommodations, and time, compared with FTF clinic visits. Moreover, telemedicine clinics appear to provide similar levels of patient satisfaction as FTF clinics in terms of medical consultations, session time, patient-physician dialogue, encouragement, and ease of communication. Telemedicine appears to represent a new trend in Saudi Arabian healthcare, and it supports Vision 2030 of the Kingdom of Saudi Arabia, for national transformation. All the aforementioned opportunities for telemedicine clinics are useful for chronic disease patients who require frequent routine follow-up care.
